# A robust mouse liver organoid platform enables sustained multicellular maturation and fibrosis modeling from a single tissue sample

**DOI:** 10.1038/s41598-026-42990-2

**Published:** 2026-03-19

**Authors:** Yingyu Liang, Yongqin Ye, Hua Xie, Vincent Chi Hang Lui, Yan Chen, Paul Kwong Hang Tam

**Affiliations:** 1 School of Pharmacy, Faculty of Medicine, Macau University of Science and Technology, Macau, 999078 China; 2 Faculty of Medicine, Macau University of Science and Technology, Macau, 999078 China; 3 Department of Pediatric Surgery, Children’s Hospital of Nanjing Medical University, Nanjing, 210093 China; 4 Department of Surgery, Li Ka Shing Faculty of Medicine, The University of Hong Kong, Hong Kong, 999077 China; 5 Precision Regenerative Medicine Research Centre, Medical Sciences Division, Macau University of Science and Technology, Macao, 999078 China

**Keywords:** Liver organoid platform, Hepatic stellate cell, A single liver tissue, Liver fibrosis modeling, Biotechnology, Cell biology, Diseases, Medical research, Stem cells

## Abstract

Efficient isolation and culture of liver organoids are critical for studying liver fibrosis, liver regeneration and drug toxicity and screening. However, preserving mature hepatobiliary characteristics and concurrently incorporating fibrosis-producing hepatic stellate cells (HSCs) remains a significant challenge, often hindering the large-scale production of organoids capable of replicating key liver functions. Here, we report a robust 3D organoid culture system that enables simultaneous isolation and long-term propagation of primary hepatocytes, cholangiocytes, and HSCs from a single source of mouse liver tissue. By supplementing the Hep-Med with Notch signaling inhibitor and dexamethasone, we achieved sustained organoid maturity, including stable albumin production, metabolic activity, and liver-specific gene expression, over multiple passages in culture. Quiescent HSCs within the system retained lipid droplets and could be activated into a myofibroblast-like phenotype, also called activated HSCs, via TGFβ stimulation. Activated HSCs impaired the proliferation and stemness, and induced epithelial-mesenchymal transition (EMT) of Hep-Orgs and Cho-Orgs, enabling in vitro liver fibrosis modeling. Optimized for minimal tissue input, this platform maximizes tissue utilization efficiency while preserving the liver’s heterogeneous cellular architecture. Its versatility supports diverse applications in liver disease modeling, drug discovery, and regenerative medicine.

**Supplementary Information:**

The online version contains supplementary material available at 10.1038/s41598-026-42990-2.

## Introduction

The liver, a central hub for metabolism and detoxification, is persistently vulnerable to diverse pathological insults. Globally, the prevalence of hepatic diseases—such as metabolic dysfunction-associated steatotic liver disease (MAFLD)—and biliary disorders, including cystic fibrosis-associated cholangiopathy, continues to rise alarmingly^1,2^. Current clinical strategies, spanning pharmacotherapy to liver transplantation, face critical limitations: donor shortages, suboptimal efficacy in advanced disease, and procedural risks^3^. These unresolved challenges underscore the pressing need for innovative therapeutic solutions.

In recent years, organoids—self-organizing 3D cultures capable of recapitulating organotypic features in vitro^4^—have emerged as transformative tools for studying tissue physiology, disease mechanisms, and drug responses^5^. These systems leverage the self-assembly potential of pluripotent stem cells, adult stem cells, or tissue-derived progenitor cells, enabling long-term expansion with preserved genetic stability^6^. Notably, human tissue-derived organoids retain donor-specific genetic and phenotypic signatures, thereby faithfully mirroring the physiological and pathological traits of their native cell sources^4,7^.

Huch et al. pioneered systems for generating cholangiocyte organoids (Cho-Orgs) from Lgr5^+^^8^ or EpCAM^+^^9^ liver cells, enabling long-term expansion and in vitro differentiation into hepatocyte-like cells (HLCs). These HLCs exhibit functional traits such as albumin (ALB) secretion and liver-specific metabolic activities. However, differentiated Cho-Orgs retain persistent cholangiocyte marker expression, reflecting incomplete hepatocyte maturation, and lose proliferative capacity after limited passages^6^. In contrast, hepatocyte organoids (Hep-Orgs) demonstrate advanced hepatic maturation, closely mirroring primary hepatocytes (Pri-Hep) in marker expression and functional profiles^10^. Recent advances in expandable Hep-Orgs cultures involve embedding primary hepatocytes in Matrigel, yielding organoids morphologically distinct from biliary progenitor-derived systems^10,11^.

In this study, we developed a refined 3D organoid platform that enables simultaneous isolation and expansion of primary hepatocytes, cholangiocytes, and hepatic stellate cells (HSCs) from a single murine liver sample. Supplementation with a Notch signaling inhibitor (DAPT) and dexamethasone sustained mature hepatic phenotypes across serial passaging, as demonstrated by persistent albumin secretion, metabolic functionality, and stable expression of hepatocyte-specific genes. Quiescent HSCs within the system retained lipid storage capacity and underwent TGFβ-responsive fibrogenic activation. Co-cultured with activated HSCs, Hep-Orgs and Cho-Orgs showed lower proliferation and stemness, and higher level of EMT, providing a robust model for in vitro fibrosis studies. The platform operates efficiently with minimal tissue input while achieving high cellular yield and preserving the majority of the native tissue architecture. By integrating multicellular diversity, functional maturity, and disease modeling capabilities, this system serves as a versatile tool for advancing liver disease research, drug screening, and regenerative therapies.

## Results

### Murine Hep-Orgs originating from single mature hepatocytes

**Fig. 1:**
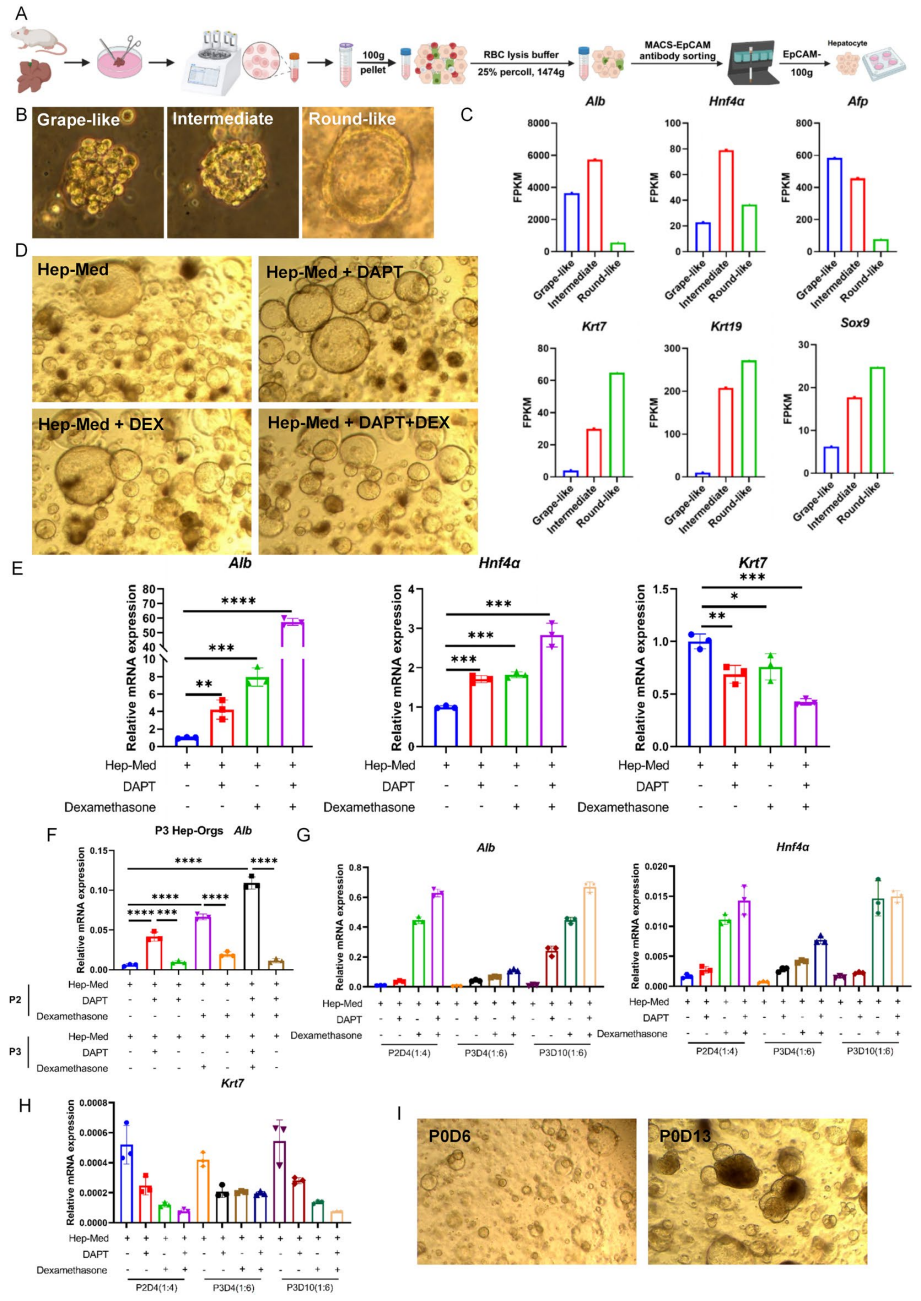
Establishment of 3D culture system of murine hepatocyte organoids. (**A**) Schematic depicting the isolation of primary hepatocytes. (**B**) Representative images of three different morphologies of Hep-Orgs at Passage 0 (P0). (**C**) Comparison of the expression of hepatocyte and cholangiocyte markers among three morphological subtypes of Hep-Orgs (P0). (**D**) Representative images of Hep-Orgs (P1) in Hep-Med supplemented with DAPT, Dex and DAPT + Dex. (**E**) qRT-PCR analysis of gene expression of hepatocyte markers and cholangiocyte markers in Hep-Orgs in different culture medium with or without DAPT, Dex and DAPT + Dex. Data are represented as mean ± SD. (**F**) qRT-PCR analysis of *Alb* expression in Hep-Orgs cultured in medium with or without DAPT, Dex and DAPT + Dex. Data are represented as mean ± SD. (**G**–**H**) qRT-PCR analysis of *Alb*, *Hnfa* and *Krt7* expression in Hep-Orgs with different ratio of passaging and different culture time points. Data are represented as mean ± SD. (**I**) Representative images of Hep-Orgs at Passage 0 (P0) in Hep-Med supplemented with DAPT, Dex.

We isolated primary hepatocytes from wild-type adult BALB/c mouse liver by combining density-gradient centrifugation with percoll solution and EpCAM-negative selection (EpCAM-) beads sorting, then suspended the cells in Matrigel™ for organoids culture (Fig. 1A). During the first passage of culture, the organoids showed three distinct morphological subtypes including grape-like, round-like and intermediate when continuously used the Hep-Medium (Hep-Med) described by Hans Clevers group^10^. (Fig. 1B). With increasing culture time, the Hep-Orgs gradually transitioned from a grape-like structure to an intermediate form and eventually grew in a round-like shape. Three subtypes of Hep-Orgs were picked out and the differentiate genes expression of was examined by Smart-Seq analysis. The results showed the markers of hepatocyte (*Alb*, *Afp*, *Hnf4α*) were higher in grape-like Hep-Orgs than the round-like shape, yet were lower for the cholangiocyte markers *Krt19*, *Krt7* or *Sox9*, and the intermediate subtype showed genes expression between other two subtypes beside *Alb*, *Hnf4α* were higher that of grape-like subtype (Fig. 1C). The loss of hepatocyte makers prompted us to refine culture conditions. Eventually resulting in more mature Hep-Orgs in the Hep-Med with DAPT and dexamethasone (Dex). Consistent with the morphology of organoids in Hep-Med, Hep-Orgs with DAPT, Dex and DAPT + Dex showed round-like appearance (Fig. 1D). The organoids in Hep-Med showed increased levels of *Alb*, *Hnf4α* and reduced *Krt7* (Fig. 1E) indicated a more mature Hep-Orgs. To further confirm the long-term use of DAPT and Dex was needed, we removed these two molecules from the third passage of culture, the expression of *Alb* markedly reduced (Fig. 1F) suggested the importance for DAPT and Dex in maintaining the expression of ALB. Interestingly, the organoids could be passaged by mechanical disruption at a ratio of 1:4 every 4–7 days to obtain the high-*Alb* mature Hep-Orgs, but not at a higher dilution such as 1:6 which the expression of *Alb*, *Hnf4α* were reduced, while the *Krt7* was increased (Fig. 1G–H). Compared to the P0 organoids in Hep-Med, the Hep-Orgs in Hep-Med with DAPT and Dex consistently maintained a rounded morphology throughout the culture period, which corresponds to the disappearance of the other two morphological subtypes (Fig. 1I).

### Hep-Orgs retain gene expression profiles of hepatocytes

**Fig. 2:**
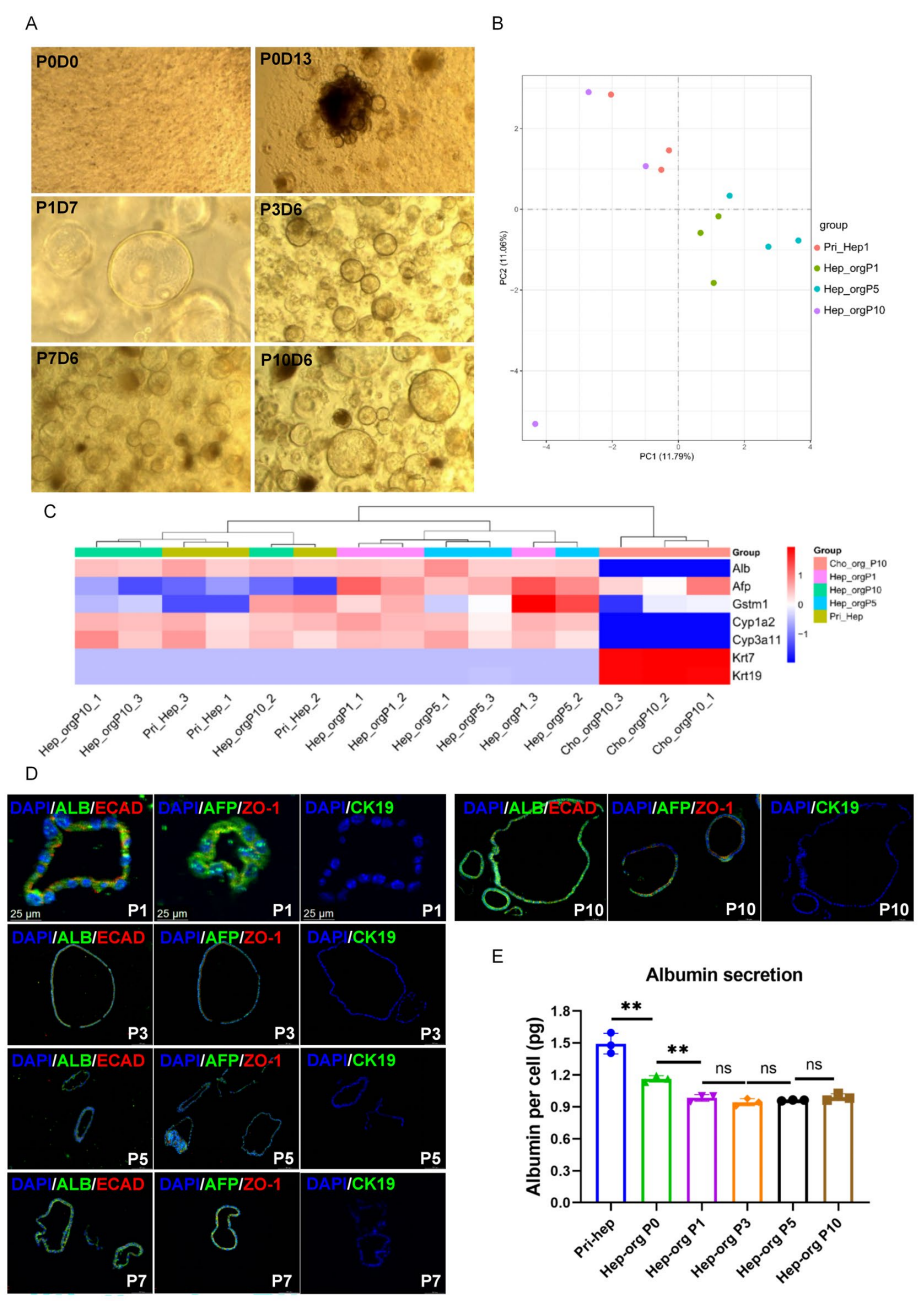
Characterization of mouse hepatocyte organoids. (**A**) Representative images of different passages of Hep-Orgs (P1, P5, P10). (**B**) PCA plot showing the clustering of Hep-Orgs at different passages (P1, P5, P10). (**C**) Heatmap of major hepatic markers in Pri-Hep, Hep-Orgs and Cho-Orgs (from three mouse labeled with _1, _2, _3, Passage 1, 5, and 10 is labeled as P1, P5 and P10). (**D**) Immunofluorescence staining of ALB, AFP, ZO-1 and CK19 of Hep-Orgs. (**E**) Albumin secretion measured after 24 h culturing of primary hepatocytes, Hep-Orgs of P0 day 13, P1, P3, P5 and P10. Data are represented as mean ± SD.

We further examined the gene expression profiles in long-term culture of Hep-Orgs in the Hep-Med supplemented with DAPT and Dex. The morphology of the organoids showed a round-like structure from passage 0 (P0) to P10 (Fig. 2A). Bulk mRNA sequencing was performed for Hep-Orgs and cholangiocytes organoids (Cho-Orgs) from mice (*n* = 3) and compared to their primary hepatocytes. The results showed the Hep-Orgs remained remarkably similarity over time as visualized by PCA plot and assessed at P1, P5 and P10 (Fig. 2B). The heatmap of main hepatic markers such as *Alb*, *Cyp1a2*, *Cyp3a11* indicated their stable expression during Hep-Orgs passaging, while *Krt7* and *Krt19* were not re-expressed (Fig. 2C). Hep-Orgs were then analyzed by immunofluorescence staining. Hep-Orgs showed strong albumin (ALB), and AFP expressions yet were negative for the cholangiocyte markers CK19 (Fig. 2D). Albumin secretion of Hep-Orgs (P0 and P1) supernatant of Hep-Orgs culture was slightly lower compared to primary hepatocytes (Pri-hep) and remained stable at P1, P3, P5 and P10 (Fig. 2E).

### Hep-Orgs retain key functions of hepatocytes

**Fig. 3:**
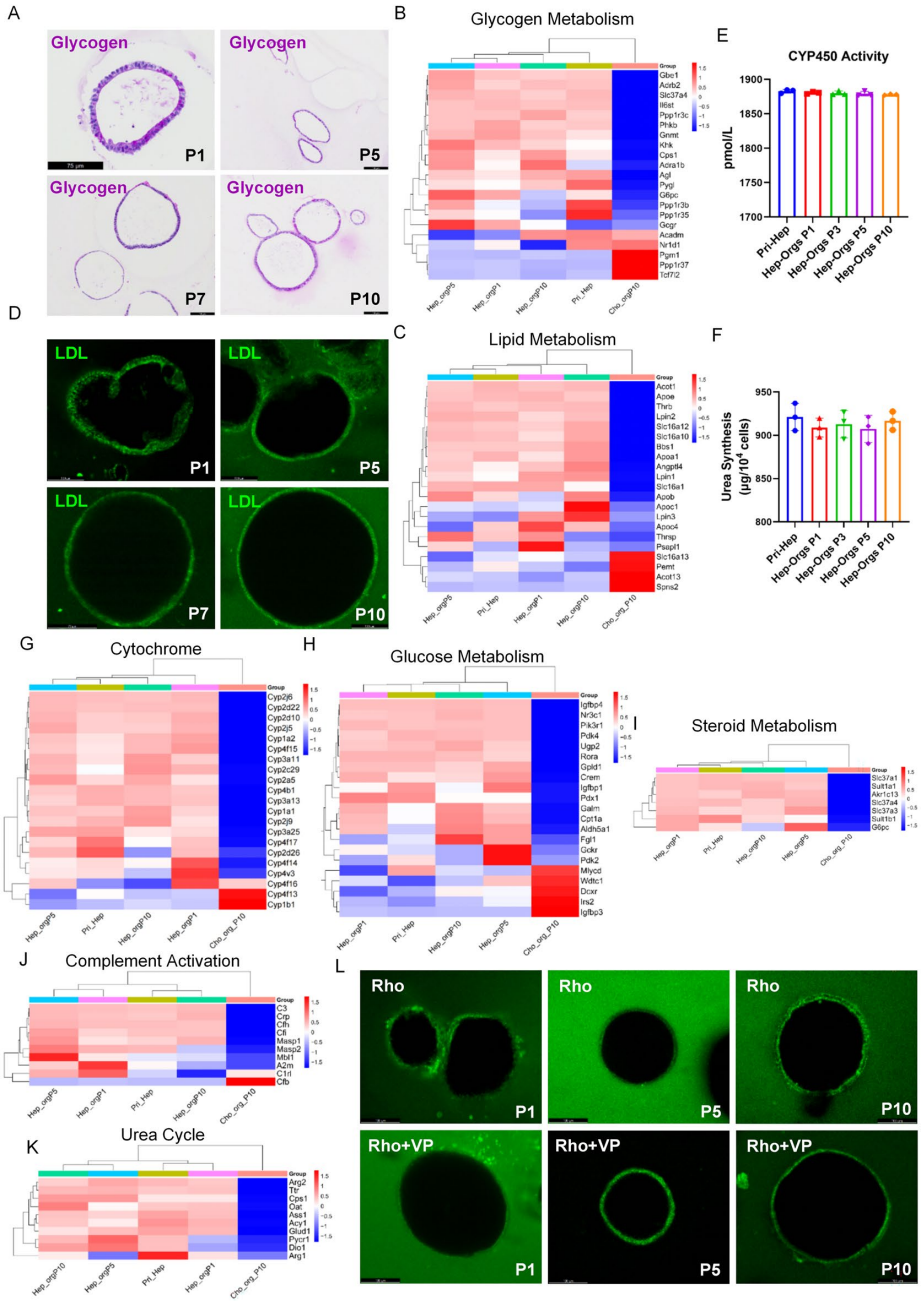
Function detection of Hepatocyte organoids. (**A**) Glycogen accumulation evaluated by Periodic-Acid Schiff (PAS) staining (dark pink) in Hep-Orgs with passage 1–10. Nuclei were stained with hematoxylin (blue). Scale bar showed 75 μm (left upper) and 100 μm (others). (**B**–**C**) Heatmaps comparing Hep-Orgs with primary hepatocytes and Cho-Orgs. glycogen metabolism (**B**) and lipid metabolism (**C**). (**D**) Low density lipoprotein (LDL) uptake was analyzed by DiO-ac-LDL fluorescent staining in cultured Hep-Orgs. (**E**) CYP450 activity measured after 24 h culturing of primary hepatocytes, Hep-Orgs of P0 day 13, P1, P3, P5 and P10. Data are represented as mean ± SD. (**F**) Urea production measured after 24 h culturing of primary hepatocytes, Hep-Orgs of P0 day 13, P1, P3, P5 and P10. Data are represented as mean ± SD. (**G**–**K**) Heatmaps comparing Hep-Orgs with primary hepatocytes and Cho-Orgs. cytochrome activity (**E**), glucose metabolism (**F**), steroid metabolism (**G**), complement activation (**H**) and urea cycle (**I**). (**L**) Representative images demonstrating the Hep-Orgs could not transport the Rhodamine 123 (Rho) into the lumen.

Hep-Orgs showed strong periodic acid-Schiff (PAS) staining indicative of glycogen accumulation (Fig. 3A) which can be observed from all stages of the culture from P1 to P10. Genes involved in hepatocyte functions such as glycogen metabolism and lipid metabolism displayed similar expression profiles between Hep-Orgs and primary hepatocytes (Fig. 3B–C). Fluorescent labeled low-density lipoprotein uptake (LDL) was readily visualized by fluorescence imaging (Fig. 3D). Similar to primary hepatocytes, Hep-Orgs showed hepatocyte functions such as Cytochrome P450 activity, glucose metabolism, steroid metabolism, complement activation and urea cycle (Fig. 3E–K). Even if it is morphologically similar to Cho-Orgs, the Hep-Orgs could not pump Rhodamine 123 (Rho) into the lumen of organoids (Fig. 3L).

### Murine functional Cho-Orgs originating from single EpCAM^+^ cells

**Fig. 4:**
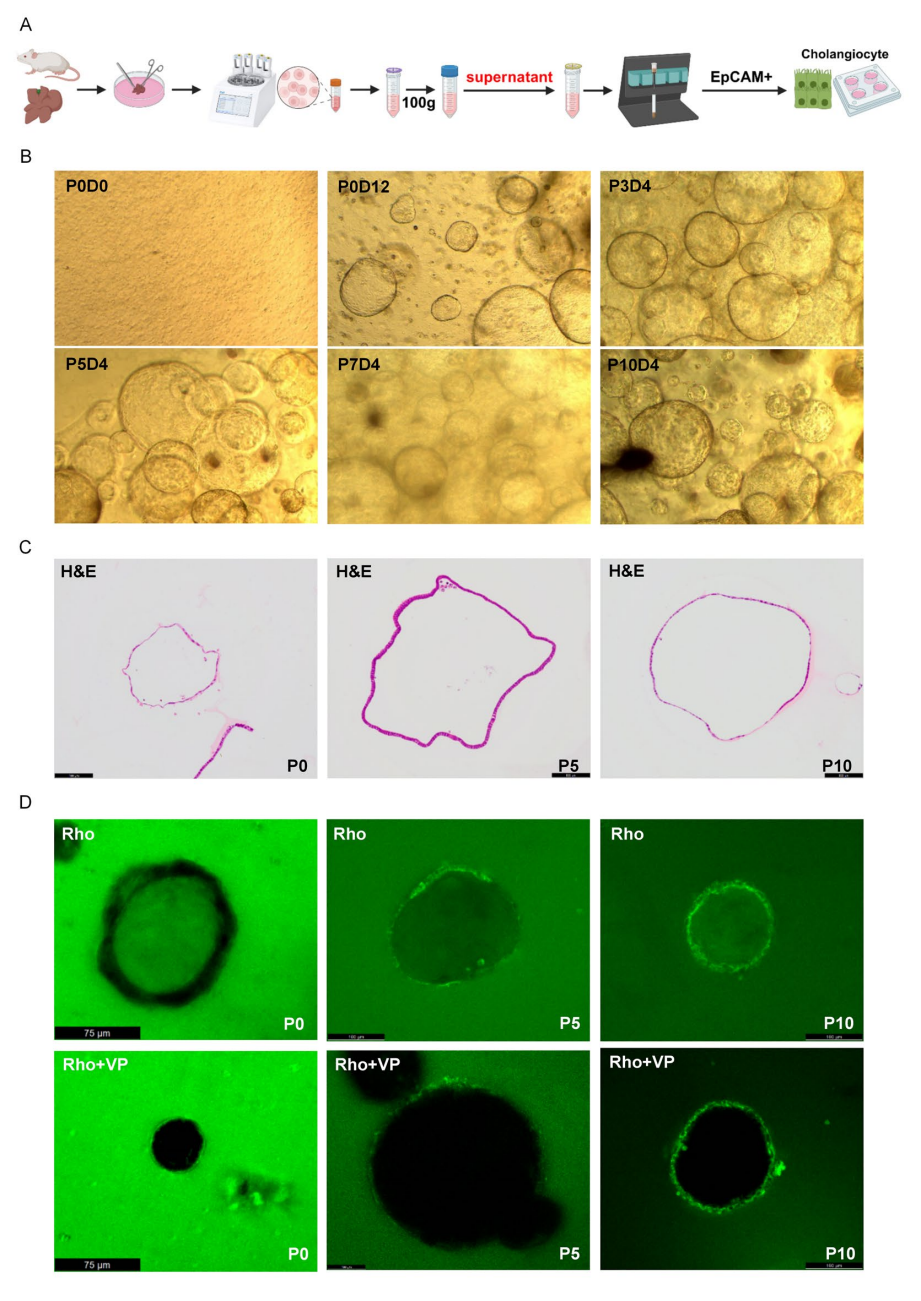
Establishment of 3D culture system of murine cholangiocyte organoids. (**A**) Schematic depicting the isolation of cholangiocytes. (**B**) Representative images of different passages of Cho-Orgs (P0-P10). (**C**) Paraffin sections of Cho-Orgs H&E-stained. Scale bar showed 100 μm. (**D**) Representative images demonstrate the inhibition of MDR1 fluorescent substrate Rhodamine 123 (Rho) by Verapamil (VP).

EpCAM^+^ cells were isolated from wild-type adult BALB/c mouse liver by EpCAM beads sorting and suspended the cells in Matrigel (Fig. 4A). Typically, Cho-Orgs showed the single-layered epithelial compartment that resembles the hepatic ductal compartment from P0 to P10 (Fig. 4B) verified by H&E staining (Fig. 4C). To test the activity of multidrug resistance protein 1b (MDR1b), which encodes a transmembrane export pump in cholangiocytes and transports compounds into the lumen of Cho-Orgs, we added the MDR1b fluorescent substrate Rho to the culture medium. In the presence of verapamil (VP), which blocks MDR1b activity, as expected, there was no accumulation of Rho in the Cho-Orgs (Fig. 4D).

### Cho-Orgs maintain gene expression profiles of cholangiocytes

**Fig. 5:**
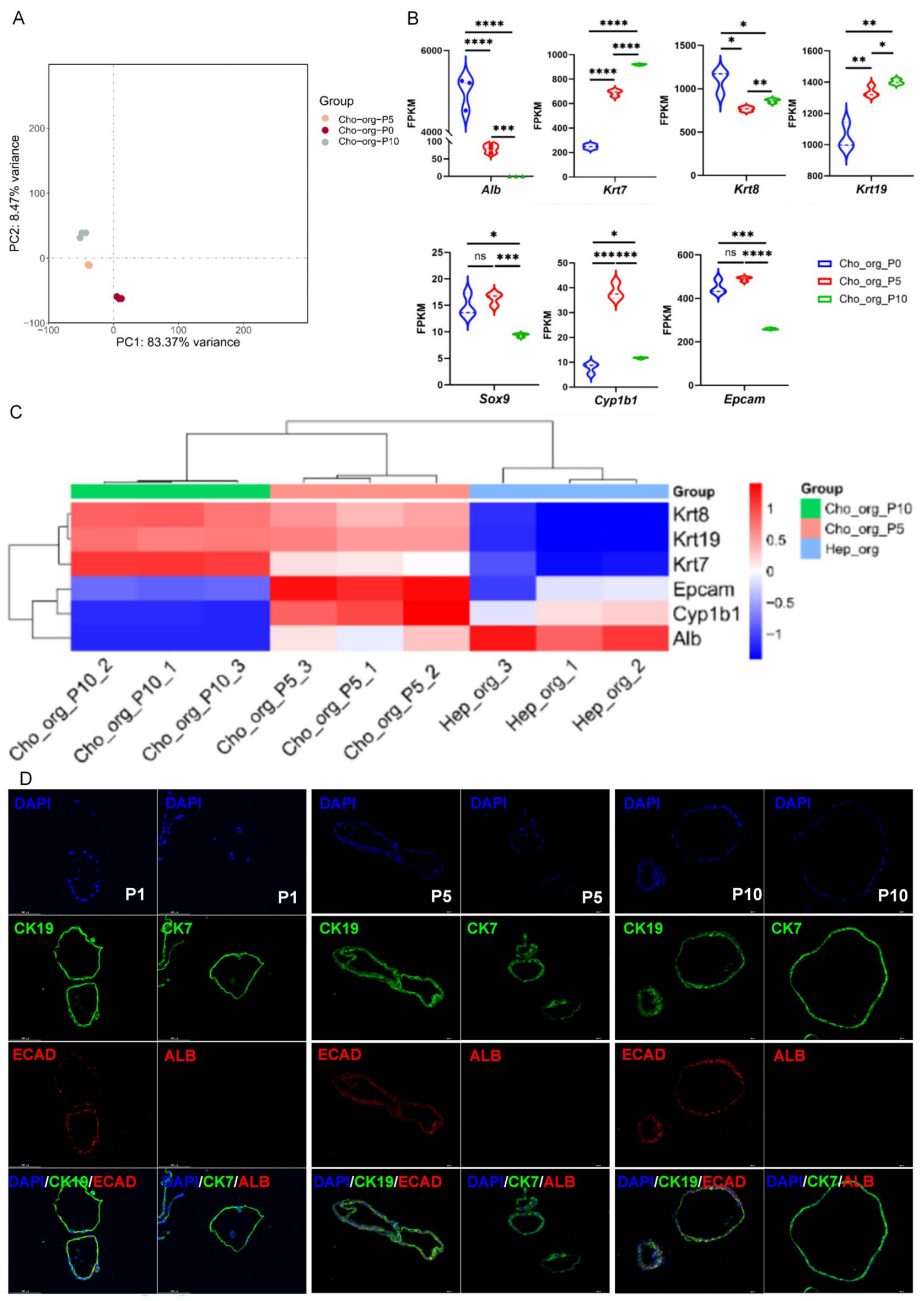
Characterization of mouse cholangiocyte organoids. (**A**) PCA plot showing the clustering of Cho-Orgs at different passages (P0, P5, P10). (**B**) Comparison of the expression of hepatocyte and cholangiocyte markers among different passages of Cho-Orgs (P0, P5, P10). (**C**) Heatmap of major cholangiocyte markers in Cho-Orgs and Hep-Orgs (from three mouse labeled with _1, _2, _3, Passage 1, 5, and 10 is labeled as P1, P5 and P10). (**D**) Representative images of immunofluorescence staining of CK19, CK7, ECAD and ALB of Cho-Orgs.

Gene expression patterns over time were assessed by mRNA sequencing for three murine Cho-Orgs cultures. *Alb* expression was strong in P0 Cho-Orgs, while markedly reduced during passaging and P10 was negative for *Alb* (Fig. 5A). Notably, the cholangiocyte maker *Krt7* and *Krt19* gradually increased and reduced immature marker *Sox9* was observed (Fig. 5B). These all indicated we obtained more mature Cho-Orgs during passaging. The heatmap of main cholangiocyte markers such as *Krt7*, *Krt8* and *Krt19* indicated their stable expression during Cho-Orgs passaging, while *Alb* were not re-expressed (Fig. 5C). Cho-Orgs were then verified by immunofluorescence staining. The organoids (P1, P5, P10) stably expressed high CK19 and CK7 expressions, while were negative for hepatocyte marker ALB (Fig. 5D). In Cho-Orgs, E-cadherin (ECAD), as a typical epithelial cadherin, exhibited continuous and strong membrane-localized expression, outlining the apical boundaries of the lumen (Fig. 5D).

### High-purity isolation of murine hepatic stellate cells

**Fig. 6:**
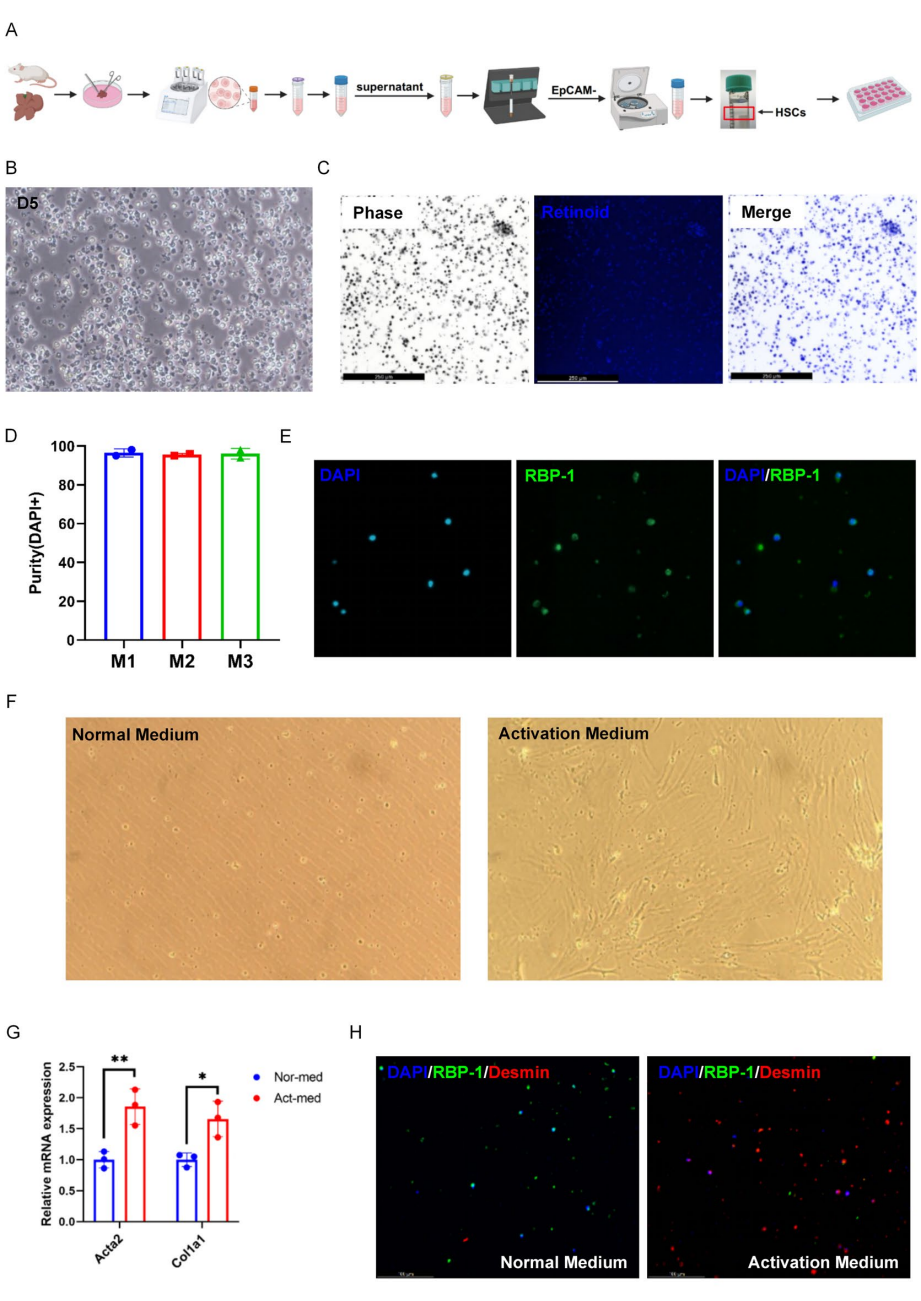
Establishment of high-purity of mouse hepatic stellate cells. (**A**) Schematic depicting the isolation of hepatic stellate cells. (**B**) Representative images of hepatic stellate cells. (**C**) Representative images of freshly isolated HSCs visualized using phase contrast microscopy (left) and retinoid fluorescence (center). A merge (right) of the retinoid fluorescence with the phase contrast image shows complete overlap of retinoid signal with characteristic lipid droplets. (**D**) Purity of HSCs derived from 3 mice labeled as M1, M2, M3. Data are represented as mean ± SD. (**E**) Immunofluorescence staining of RBP-1 of hepatic stellate cells. (**F**) Representative images of HSCs cultured with activation medium including TGFβ. (**G**) qRT-PCR analysis of *Acta2* and *Col1a1* expression in HSCs cultured with activation medium including TGFβ. Data are represented as mean ± SD. (**H**) Immunofluorescence staining of RBP-1 and Desmin of TGFβ-treated HSCs.

We isolated hepatic stellate cells (HSCs) from EpCAM-negative cells suspension by density-gradient centrifugation with Nycodenz^®^ solution (Fig. 6A). Quiescent HSCs showed the typical lipid droplets called retinoids in the cytoplasm (Fig. 6B). Retinoids are subject to rapid bleaching, and brief exposure of HSCs to UV light completely abrogates HSC fluorescence in the DAPI channel providing evidence of HSCs high-purity (> 95%) (Fig. 6C-D). Immunofluorescence staining was also performed to detect the retinoids (Fig. 6E). The HSCs showed positive expression of retinoid binding protein 1 (RBP-1) (Fig. 6E). To test the function of HSCs, we used activation medium supplemented with TGFβ to culture HSCs for 5 days. HSCs transitioned from an irregular shape containing lipid droplets to a myofibroblast-like form (Fig. 6F). The myofibroblast marker *Acta2* and *Col1a1* were examined by qPCR and the results showed upregulated expression in the TGFβ-supplemented group (Fig. 6G). Immunofluorescence staining also showed HSCs treated with TGFβ expressed increased fibrotic marker Desmin and reduced quiescent marker RBP-1 (Fig. 6H).

### Activated HSCs impaired proliferation and stemness, and induced EMT of Hep-Orgs

**Fig. 7:**
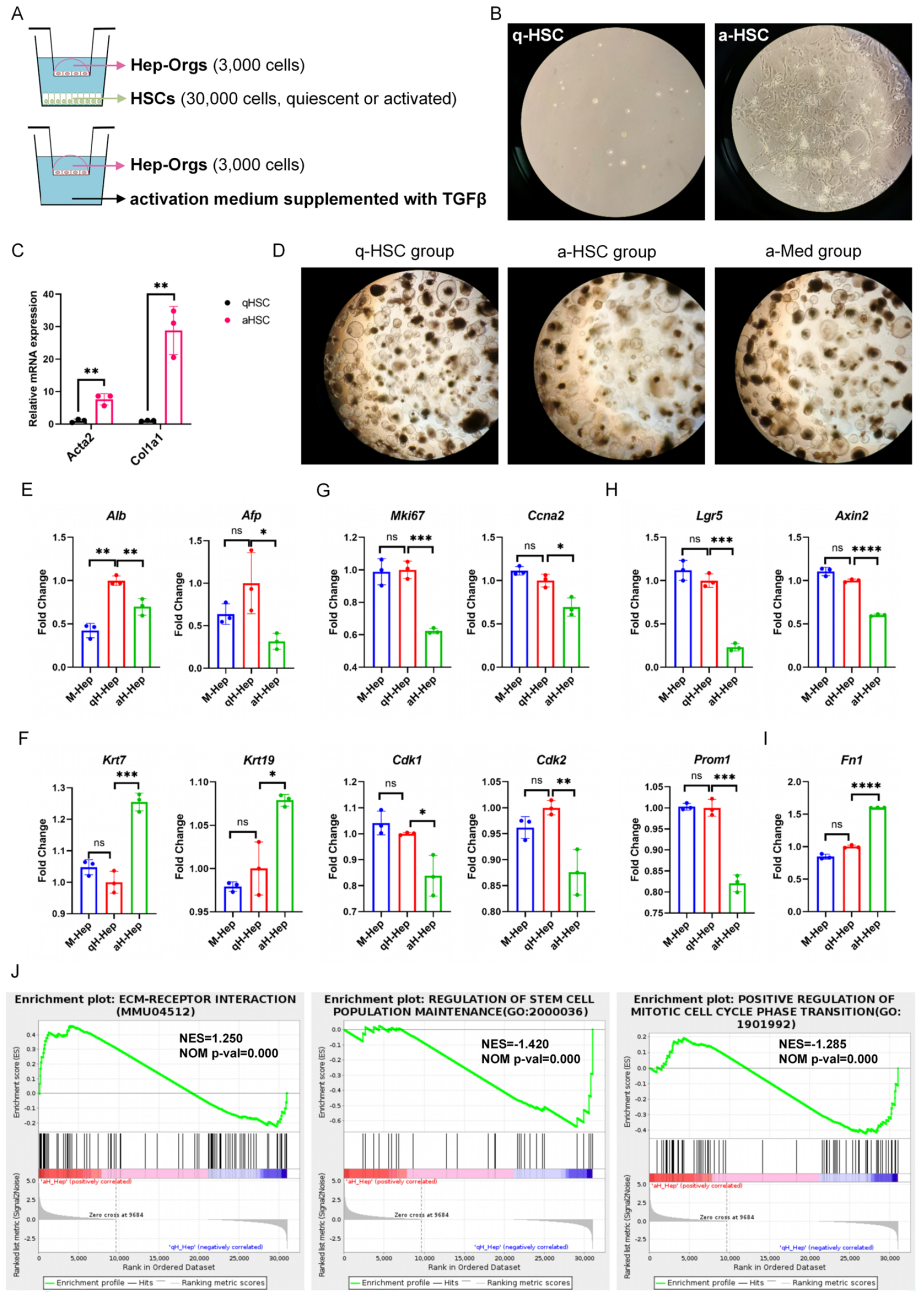
Activated HSCs hampered proliferation and stemness, and induced EMT of Hep-Orgs. (**A**) Schematic representation of co-culture system. (**B**) Representative images of quiescent and activated HSC. (**C**) qRT-PCR analysis of *Acta2* and *Col1a1* expression in quiescent and activated HSC. (**D**) Representative images of Hep-Orgs among 3 groups (q-HSC, a-HSC and a-Med). (**E**–**I**) qRT-PCR analysis after co-culturing for 14 days of hepatocyte (**E**), cholangiocyte (**F**), proliferation and cell cycle (**G**), progenitor (**H**) and EMT markers (**I**). (**J**) GSEA analysis of Hep-Orgs between q-HSC and a-HSC group.

To further test the function of activated HSCs in our system, they were co-cultured with Hep-Orgs in a non-contact transwell system for 14 days, which allows for the exchange of soluble factors (Fig. 7A). Activated by TGFβ for 10 days, quiescent HSCs (q-HSC) transitioned to a myofibroblast-like form, called activated HSC (a-HSC), with higher expression of *Acta2* and *Col1a1* (Fig. 7B–C). Notably, the Hep-Orgs in a-HSC group induced by activation medium (a-Med) showed a slower growth rate and smaller size (Fig. 7D). We further examined the gene expression profiles among 3 groups. Co-cultured with a-HSC, Hep-Orgs showed lower expression of hepatocyte makers (*Alb* and *Afp*) (Fig. 7E), while expressing stronger cholangiocyte marker (*Krt7* and *Krt19*) (Fig. 7F). Moreover, their proliferation and stemness were impaired, exhibiting low level of *Mki67*, cell cycle markers (*Ccna2*, *Cdk1* and *Cdk2*) (Fig. 7G) and progenitor markers (*Lgr5*, *Axin2* and *Prom1*) (Fig. 7H). Importantly, a-HSC induced higher expression of *Fn1* of the Hep-Orgs, indicating epithelial-mesenchymal transition (EMT) induction. (Fig. 7I). Gene Set Enrichment Analysis (GSEA) results also showed that ECM-receptor interaction pathway was upregulated, while regulation of stem cell population maintenance and positive regulation of mitotic cell cycle phase transition pathways were inhibited in Hep-Orgs co-cultured with a-HSC (Fig. 7J). The similar effects could be observed in the Cho-Orgs co-cultured with a-HSC (Data not shown).

## Discussion

Current liver organoid systems face critical limitations including (1) inability to maintain mature hepatobiliary functions while incorporating fibrosis-competent HSCs, and (2) inefficient cell isolation from limited clinical specimens. Our 3D platform overcomes these challenges by simultaneously isolating hepatocytes, cholangiocytes, and HSCs from minimal murine tissue while preserving native cellular status. The Notch inhibitor/dexamethasone cocktail sustains hepatocyte maturity (albumin secretion, metabolic activity) across passages, overcoming functional decline in conventional models. Crucially, quiescent HSCs retain lipid storage and activate into fibrotic phenotypes upon TGFβ exposure, enabling integrated studies of homeostasis and disease. With efficient tissue utilization, this system bypasses scalability barriers while mirroring human liver complexity which directly addresses roadblocks in drug testing and regenerative therapy development.

In Huch’s system^10^, primary hepatocyte isolation relies on a technically demanding workflow involving two-step collagenase perfusion, low-speed centrifugation, and Percoll gradient purification. Similarly, Clevers^12^ and colleagues employed collagenase type IV digestion paired with manual tissue fragmentation to procure hepatocytes. We identified critical limitations in these established protocols, including compromised cell viability, high procedural failure rates, and an inability to concurrently isolate cholangiocytes and HSCs. Our optimized approach streamlines this process: enzymatic dissociation generates single-cell suspensions, followed by sequential Percoll density gradients and EpCAM-based magnetic sorting. This protocol enables simultaneous isolation of hepatocytes, cholangiocytes, and HSCs with high purity, addressing key bottlenecks in multi-lineage liver cell procurement.

The Notch signaling pathway, an evolutionarily conserved regulator, governs embryonic development, cellular differentiation, and stem cell self-renewal by maintaining “stemness” properties^13,14^. During hepatogenic differentiation of mesenchymal stem cells (MSCs), Notch activity declines^15^, reflecting its role in balancing lineage commitment across tissues—including liver, immune, and gastrointestinal systems^16–20^. Pharmacological Notch inhibition via DAPT drives hepatocyte maturation by upregulating hepatocyte-specific genes/proteins (e.g., albumin) while suppressing cholangiocyte differentiation through HNF-1β downregulation^20,21^. While Hu et al.^9^ achieved transient hepatocyte-like cell differentiation using sequential DAPT (days 1–9) and dexamethasone (days 10–12), our study demonstrates that continuous Notch inhibition is critical for sustaining both molecular (e.g., albumin stability) and functional maturation in hepatic organoids.

Glucocorticoids, particularly dexamethasone, are essential for maintaining viability and liver-specific functionality in primary hepatocyte cultures^22,23^. Dexamethasone enhances albumin (ALB) expression in hepatocytes^24^ and confers protection against apoptosis via cFLIP-mediated inhibition of death receptor signalling^25^. In our system, sustained supplementation of Hep-Med with DAPT and dexamethasone synergistically elevated ALB levels in hepatocyte organoids (Hep-Orgs), whereas dexamethasone withdrawal resulted in paradoxical ALB suppression—highlighting distinct regulatory mechanisms for these agents. Despite this combinatorial strategy, Hep-Orgs ALB expression remains inferior to primary hepatocytes and the miner different on metabolism-related gene expression, emphasizing unresolved gaps in hepatocyte-specific gene regulation that require mechanistic elucidation to advance translational utility. Moreover, primary hepatocytes in standard 2D monolayer cultures rapidly lose expression of liver-specific genes, including ALB, within just a few days of isolation^26^, while Hep-Orgs in our system maintain the characteristics of primary hepatocytes after passaging. Additionally, we initially established the hepatocyte organoid based on the published Huch’s protocol^10^, and also exhibited both periportal and pericentral metabolic functions as theirs. However, distinct hepatocyte subpopulations are spatially segregated along the portal-central axis and are critical to understanding metabolic homeostasis^27^. Hasan’s team established multi-zonal organoid system serving as an in vitro human model to better recapitulate hepatic architecture relevant to liver development and disease^28^. Moreover, the 3D air-liquid interface (3D-ALI)^29,30^ method on liver slices can be used to distinguish different hepatocyte subpopulations based on their spatial distribution.

Our system highlights the isolation of 3 cell types from a single liver and maximizes tissue utilization efficiency and preserves the liver’s heterogeneous cellular architecture, thereby further facilitating the simultaneous investigation of pathological features in three distinct cell types from the same diseased sample. However, our platform does not replicate the intricate cellular composition and tissue architecture observed in vivo, rendering it unsuitable for investigations into bile canaliculi–bile duct cell–cell interactions and architectural organization. Recently established multi-lineage liver platforms^31^ can mimic the cellular interactions of the periportal region.

Hepatic fibrosis is driven by the activation of quiescent HSCs, mediated through crosstalk with liver-resident and infiltrating immune cells^32–34^. Lineage-tracing studies confirm HSCs as the primary source of myofibroblasts (82–96% in chronic liver injury models)^35^, underscoring the need for reliable isolation of high-purity quiescent HSCs. While Schwabe’s protocol^36^ combines in situ/in vitro digestion with Nycodenz^®^ gradient centrifugation, its complexity compromises cell viability and yields inconsistent results. Our streamlined method isolates HSCs from EpCAM-negative cell suspensions via Nycodenz^®^ density-gradient centrifugation, minimizing cell loss and mortality associated with multi-step enzymatic digestion. Following TGFβ stimulation, these HSCs undergo phenotypic transition from vitamin A-rich pericytes to ECM-secreting myofibroblasts, also called activated HSCs (a-HSCs). Hepatic fibrosis is characterized by the formation of fibrous scars due to the excessive accumulation of extracellular matrix (ECM) proteins, primarily cross-linked type I and type III collagens, which replace damaged normal tissue^37^. Regulation of liver fibrosis is complex and involves crosstalk between resident non-parenchymal and parenchymal cells^38^. Importantly, HSCs activation is a critical step in preventing the development of fibrosis^39^. Activated HSCs migrate to the site of injury and secrete ECM to produce a fibrous scar^40^. Previous studies showed HSCs also affected hepatocyte and cholangiocyte proliferation and EMT through paracrine signaling mechanisms in liver fibrosis, including hepatocyte growth factor (HGF), TGF-β1 and interleukin-6 (IL-6)^41–43^. In our system, we initially established normal hepatocyte and cholangiocyte organoids to assess the alterations of parenchymal cells in the co-culture system. HSCs supplemented with activation medium exhibited the reduced RBP-1 expression, q-HSCs marker, and elevated *Acta2*, *Col1a1* and Desmin, indicating successful activation of HSCs, similar to the a-HSCs observed in vivo in liver fibrosis. In the co-culture system, a-HSCs induced impaired proliferation, stemness, and EMT of Hep-Orgs and Cho-Orgs. These effects mirrored the phenotypic changes seen in vivo during liver fibrosis, supporting the utility of this system for robust in vitro fibrosis modeling. It should be noted that while our study provides robust evidence for TGFβ-mediated HSCs activation, the experimental framework was primarily designed to elucidate this specific pathway. Other clinically relevant mechanisms contributing to fibrosis pathogenesis—particularly inflammatory cascades and immune-mediated processes—were beyond the scope of the current model. Future investigations employing co-culture systems or in vivo models would be valuable to explore these complementary mechanisms.

Our culture method enables the simultaneous generation of hepatocyte organoids, cholangiocyte organoids, and hepatic stellate cells from a single mouse liver tissue source, maximizing tissue efficiency while preserving native cellular heterogeneity. This unified platform supports diverse applications—from disease modeling and drug screening to regenerative medicine. Future studies should validate this approach in human liver samples to advance its translational potential.

## Methods

### Animal models

BALB/c mice were purchased from Zhuhai Bestest Biotechnology Co., Ltd. and housed in the Macau University of Science and Technology specific pathogen-free animal facility at 20–22 °C and 30–70% humidity with a 12-h light/12-h dark cycle. All experiments were conducted using 6- to 8-week-old mice weighing approximately 22 g. All animal experiments were performed after being reviewed and approved by the Animal Ethics Committee of the Macau University of Science and Technology (MUST-FDCT-20241114001). All experimental procedures were carried out in strict compliance with the ARRIVE (Animal Research: Reporting of In Vivo Experiments) Guidelines (PLoS Bio 8(6), e1000412, 2010) to ensure the ethical and accurate reporting of animal research and this study was conducted in accordance with the relevant guidelines. The sacrificing procedure was performed under general anesthesia induced by intraperitoneal injection of 1.25% tribromoethanol (Sigma, 75-80-9), followed by cervical dislocation.

### Isolation of hepatocytes, cholangiocytes and hepatic stellate cells

#### Steps for hepatocyte organoids

Liver tissue was collected from mice, minced, and digested in a Single Cell Tube containing 5 mL of digestion medium (see key resources table) using the Mouse_Liver_Heater mode. Following enzymatic digestion termination, 5 mL of wash buffer (99% ice-cold advanced DMEM/F12 basal medium, 1% Penicillin/Streptomycin) was added to stop the reaction. The cell suspension was filtered through a 100 μm cell strainer and centrifuged at 100 g (4 °C) for 5 min. The pellet containing hepatocytes for further purification was retained, while the supernatant (namely SUPERNATANT A) was transferred to a new tube for subsequent isolation of cholangiocytes and hepatic stellate cells. The hepatocytes pellet was treated with 1 mL red blood cell lysis buffer with thorough mixing, followed by 3–5 min incubation on ice. The reaction was terminated by adding 4 mL wash buffer, followed by centrifugation at 100 g (4 °C) for 5 min. After supernatant removal, the pellet was resuspended in 5 mL wash buffer. A discontinuous density gradient was prepared by underlaying 15 mL of 25% Percoll solution in a fresh centrifuge tube. The cell suspension was carefully layered onto the Percoll gradient, creating visible phase separation. Gradient centrifugation was performed at 1,474 g (4 °C) for 20 min. The resulting cell pellet was resuspended in 1 mL ice-cold advanced DMEM/F12 basal medium for cell counting. After subsequent centrifugation, the supernatant was discarded, and the cells were suspended in 100 µL column buffer. CD326 magnetic beads were added at 10 µL per 10^7^ cells, followed by 15-minute incubation at 4 °C protected from light. The reaction stopped with 900 µL column buffer (95% autoMACS rinsing solution, 5% MACS BSA stock solution). The magnetic separation system was prepared by priming LS columns with 3 mL column buffer. The cell-bead mixture was applied to the column, followed by three sequential 3 mL washes with MACS buffer. The EpCAM-negative cell fraction was collected by centrifugation at 100 g (4 °C) for 5 min and resuspended in 2 mL ice-cold advanced DMEM/F12 medium. After another centrifugation cycle (100 g, 4 °C, 5 min), the pellet was resuspended in Matrigel™ at a 1:2000 matrix-to-cell ratio. The cell-Matrigel™ mixture was plated and allowed to polymerize before adding 500 µL hepatocyte organoids medium (Table S1) per well. More details about reagents could be found in Table S2.

#### Steps for cholangiocyte organoids

Liver-tissue derived cholangiocyte organoids were generated following our previously published protocol^44^. The SUPERNATANT A retained from steps for hepatocytes isolation was filtered through a 40 μm cell strainer and quantified. The filtered suspension was centrifuged at 580 g (4 °C) for 10 min. After supernatant (namely SUPERNATANT B) removal in to new tube, the cell pellet was resuspended in 100 µL of magnetic bead incubation buffer. CD326 (EpCAM) magnetic beads were added at a ratio of 10 µL per 10^7^ cells, followed by 15-minute incubation at 4 °C protected from light. The reaction was terminated by adding 900 µL of magnetic bead incubation buffer. The magnetic separation system was assembled with pre-washed LS columns and collection tubes for both EpCAM-negative and -positive fractions. Columns were equilibrated with 3 mL of column buffer, and the cell-bead mixture was applied as the buffer meniscus reached the column surface. Following sample application, three sequential 3 mL washes with column buffer were performed. The flow-through containing the EpCAM-negative fraction was collected for subsequent hepatic stellate cells isolation. For EpCAM-positive cell recovery, the column was removed from the magnetic separator and placed onto a fresh collection tube. Positive cells were eluted by flushing the column with a 5 mL column buffer using the plunger supplied with the column. The eluted cell suspension was centrifuged at 300 g (4 °C) for 10 min. The pellet was resuspended in 1 mL ice-cold advanced DMEM/F12 basal medium for cell counting. After another centrifugation cycle (300 g, 4 °C, 10 min), the cell pellet was resuspended in Matrigel™ at a 1:1000 matrix-to-cell ratio. The cell-Matrigel™ suspension was plated and allowed to polymerize before adding 500 µl of cholangiocyte organoid medium (Table S1) per well. More details about reagents could be found in Table S2.

#### Steps for primary hepatic stellate cells

The isolation of primary hepatic stellate cells (HSCs) follows the protocol of Mederacke et al.^36^. The EpCAM-negative cell SUPERNATANT B from cholangiocytes isolation was centrifuged at 580 g (4 °C) for 10 min. The cell pellet was resuspended in 50 mL of GBSS/B and centrifuged again at 580 g (4 °C) for 10 min. After supernatant removal, approximately 10 mL of GBSS/B was retained, and the cell pellet was resuspended with 120 µL of DNase I solution. The volume was adjusted to 50 mL with GBSS/B, followed by centrifugation at 580 g (4 °C) for 10 min. This washing procedure was repeated, retaining approximately 10 ml of GBSS/B and adding 120 µL of DNase I solution. The cell suspension was adjusted to 22 mL with GBSS/B and mixed with 16 mL of Nycodenz gradient medium. The mixture was equally distributed into four 12 mL centrifuge tubes. Tubes were gently inverted to coat the inner surfaces, and 1.5 mL of GBSS/B was carefully layered onto each sample using a 5 mL syringe. Density gradient centrifugation was performed at 1,380 g (4 °C) for 17 min using a non-braking protocol with constant acceleration and deceleration. A distinct white layer at the 12 mL interface, containing hepatic stellate cells, was collected and transferred to a fresh centrifuge tube for cell counting. Cells were plated at 100,000 cells/well in 24-well plates. Following centrifugation at 580 g (4 °C) for 10 min, the cell pellet was resuspended in complete hepatic stellate cell medium and transferred to culture plates. More details about reagents could be found in Table S2.

### Immunofluorescence staining for organoids

Organoids embedded in Matrigel™ were fixed with 4% (w/v) paraformaldehyde in phosphate-buffered saline (PBS) for 20 min at 4 °C. Following fixation, the supernatant was carefully aspirated, and 200 µL of HistoGel™ was added to facilitate paraffin embedding. Tissue sections were cut at 5 μm thickness using a microtome. For deparaffinization, sections were sequentially treated with xylene (3 × 5 min), absolute ethanol (2 × 3 min), and 95% ethanol (1 × 3 min), followed by a 1-minute rinse in running tap water. Antigen retrieval was performed using an appropriate buffer system. Non-specific binding sites were blocked with 5% bovine serum albumin (BSA) in PBS for 1 h at room temperature. Primary antibodies, diluted in 5% BSA, were applied to the sections and incubated overnight at 4 °C. After three 5-minute washes with PBS, sections were incubated with species-specific secondary antibodies conjugated to fluorescent dyes for 1 h at room temperature. Following additional PBS washes, nuclear staining was performed using 4’,6-diamidino-2-phenylindole (DAPI) for 5 min at room temperature. Finally, sections were mounted using Fluoromount-G™ aqueous mounting medium and stored at 4 °C protected from light until imaging. More details about reagents could be found in Table S2.

### H&E staining

Organoids were initially fixed in 4% paraformaldehyde (PFA) and subsequently embedded in paraffin using standard histological protocols. Paraffin-embedded Sect.  (5 μm thickness) were deparaffinized in xylene and rehydrated through a graded ethanol series (100%, 95%, and 70%). For hematoxylin and eosin (H&E) staining, sections were stained with Vector^®^ Hematoxylin for 5 min, followed by three 1-minute washes in running tap water. Nuclear staining was enhanced by treatment with Scott’s tap water (30 s) and two additional tap water rinses. Cytoplasmic counterstaining was performed using Eosin Y solution (30 s), followed by tap water rinsing. Sections were then dehydrated through an ascending ethanol series, cleared in xylene, and mounted with permanent mounting medium. More details about reagents could be found in Table S2.

### Periodic acid-schiff (PAS) staining

For PAS staining, deparaffinized sections were treated with 1% periodic acid solution for 5 min at room temperature to oxidize carbohydrate moieties. After two 5-minute washes with PBS, sections were incubated with Schiff’s reagent for 15 min to detect aldehyde groups formed during oxidation. Following two additional PBS washes, sections were counterstained with Mayer’s hematoxylin for 2 min to visualize nuclear morphology. Excess stain was removed through three 5-minute PBS washes. Stained sections were dehydrated, cleared, and mounted for microscopic examination using bright-field microscopy to assess glycosylation patterns. More details about reagents could be found in Table S2.

### Bulk RNA sequencing of organoids

A total of 1 µg total RNA per sample was used as input material for the mRNA library preparation. Most of Eukaryote ‘mRNA have polyA tails, which are enriched by Oligo (dT) magnetic beads. Then, the obtained mRNA was randomly interrupted by bivalent cations, and the first strand of cDNA was synthesized in the 1st Strand Enzymes Reverse transcriptase system using the segmented mRNA as the template and the random oligonucleotide as the primer, and the second strand of cDNA was synthesized using the 2nd Strand Enzymes and dNTPs. Purified double-stranded cDNA undergoes end repair, A-tailing, and ligation with sequencing adapters. cDNA with an insert size of approximately 200–300 bp is screened. PCR amplification is performed, and the PCR product is purified again, ultimately obtaining a library. And then, library quality was assessed on the Agilent 4200 TapeStation. After passing the library inspection, the library preparations were sequenced on a NovaSeq 6000 platform (CHI BIOTECH CO.,LTD) and 150 bp stand-specific paired-end reads were generated. The transcriptome sequencing and analysis were conducted by AccuraMed Technology Limited (Guangzhou, China). Bulk RNA-seq was performed using biological replicates​ (n = 3), where each replicate originated from an independent cell culture.

### Smart sequencing of organoids

Total RNA was extracted using the TRIzol reagent according to the manufacturer’s protocol. RNA purity and quantification were evaluated using the NanoDrop 2000 spectrophotometer. RNA integrity was assessed using the Agilent 2100 Bioanalyzer. Full-length mRNA amplification was performed using the Single Cell Full Length mRNA-Amplification Kit according to the manufacturer’s protocol. Following quality control assessment, qualified cDNA samples were used for library preparation with the cDNATruePrep DNA Library Prep Kit V2 for Illumina. The transcriptome libraries were constructed following the manufacturer’s instructions, including cDNA fragmentation, end repair, adapter ligation, and PCR amplification steps. Library quality was verified using the Agilent 2100 Bioanalyzer system before sequencing. The transcriptome sequencing and analysis were conducted by OE Biotech Co., Ltd. (Shanghai, China).

### Differentially expressed gene (DEG) and pathway enrichment analysis

Prior to differential gene expression analysis, for each sequenced library, the read counts were adjusted by edgeR program package through one scaling normalized factor. Differential expression analysis of two conditions was performed using the edgeR R package (3.40.2). The P values were adjusted using the Benjamini & Hochberg method. Corrected P-value of 0.05 were set as the threshold for significantly differential expression.

Gene Set Enrichment Analysis (GSEA) is a computational approach to determine if a pre-defined Gene Set can show a significant consistent difference between two biological states. The genes were ranked according to the degree of differential expression in the two samples, and then the predefined Gene Set were tested to see if they were enriched at the top or bottom of the list. Gene set enrichment analysis can include subtle expression changes. We used clusterProfiler R (4.22) to perform GSEA analysis on GO dataset of this species.

### Total RNA extraction and quantitative RT-PCR analysis

Total RNA was isolated from organoids using the Direct-zol™ RNA MiniPrep Kit according to the manufacturer’s protocol. RNA concentration and purity were determined spectrophotometrically using the A260/A280 ratio. Genomic DNA contamination was eliminated using the gDNA Eraser component of the PrimeScript™ RT Reagent Kit with gDNA Eraser. First-strand cDNA synthesis was performed following the manufacturer’s recommended protocol using 1 µg of total RNA as template. Quantitative real-time PCR (qPCR) analysis was conducted using iTaq™ Universal SYBR^®^ on a QuantStudio™ real-time PCR system. Reactions were performed in triplicate under the following cycling conditions: initial denaturation at 95 °C for 20 s, followed by 40 cycles of 95 °C for 3 s and 60 °C for 30 s. Melt curve analysis was performed to verify amplification specificity. Relative gene expression was calculated using the 2^−ΔΔCt^ method, with normalization to housekeeping genes. More details about reagents could be found in Table S2.

### Low density lipoprotein (LDL) uptake analysis

In this study, low-density lipoprotein (LDL) uptake and distribution were analyzed using fluorescently labeled human 3,3’-dioctadecyloxacarbocyanine perchlorate-LDL (DiO-LDL). Organoids were incubated with 10 µg/mL DiO-LDL in complete culture medium at 37 °C under 5% CO_2_ conditions for 3 h. Following incubation, organoids were washed three times with DPBS to remove unbound DiO-LDL. Fluorescence imaging was performed using a laser scanning confocal microscope equipped with a 488 nm argon laser for excitation and appropriate emission filters (500–550 nm). Z-stack images were acquired at 1 μm intervals through the organoid structures to assess spatial distribution of LDL uptake. Fluorescence intensity was quantified using image analysis software with background subtraction and normalization to untreated controls. Three independent biological replicates were performed for each experimental condition. More details about reagents could be found in Table S2.

### Rhodamine 123 assay

To determine the function of multidrug resistance protein 1B (MDR1b), Rhodamine 123 (Rho123) was performed based on our previously published protocol^44^. Briefly, cold Advanced DMEM/F12 medium was used to remove Matrigel from organoids, subsequently pretreated with DMSO as control or 10 µM Verapamil (MDR1b inhibitor) for 30 min, followed by 5 min of incubation with 100 µM Rho123. The organoids were washed 3 times with expansion medium. Fluorescence was visualized with a Leica confocal system MICA. To investigate the functional activity of multidrug resistance protein 1B (MDR1b), Rhodamine 123 (Rho123) efflux assays were performed according to our previously established protocol. Briefly, organoids were dissociated from Matrigel™ using ice-cold Advanced DMEM/F12 medium. The organoids were then pretreated for 30 min at 37 °C with either DMSO (0.1% v/v) as vehicle control or 10 µM verapamil hydrochloride, a specific MDR1b inhibitor. Following pretreatment, organoids were incubated with 100 µM Rho123 for 5 min at 37 °C in a 5% CO_2_ atmosphere. After incubation, organoids were washed three times with organoid expansion medium to remove unincorporated Rho123. Fluorescence imaging was performed using a confocal laser scanning microscope system equipped with a 488 nm argon laser for excitation and appropriate emission filters (505–550 nm). Three independent biological replicates were performed for each experimental condition. More details about reagents could be found in Table S2.

### Detection of albumin secretion

Albumin (ALB) secretion levels in organoid culture supernatants were quantified using a commercially available Mouse Albumin ELISA Quantitation Kit according to the manufacturer’s protocol. Briefly, culture supernatants were collected after 24 h of incubation and centrifuged at 300 g for 10 min at 4 °C to remove cellular debris. Standards and samples were assayed in duplicate using 100 µL aliquots. The optical density was measured at 450 nm using a microplate reader, with wavelength correction set at 630 nm. Albumin concentrations were determined by interpolation from the standard curve generated using recombinant mouse albumin standards. Three independent biological replicates were performed for each experimental condition. More details about reagents could be found in Table S2.

### Cytochrome P450 (CYP) ELISA quantification

CYP450 levels were determined using a competitive ELISA, where supernatant or standards (0.1–100 ng/mL) were incubated with HRP-conjugated anti-CYP450 antibody in pre-coated plates. After washing, TMB substrate was added, and absorbance (450 nm) was measured following acid stop. Concentrations were calculated against a standard curve (4-parameter fit), with validation confirming sensitivity (LOD 0.3 ng/mL), linearity (1–50 ng/mL), and minimal cross-reactivity (< 5% for major CYP isoforms). More details about reagents could be found in Table S2.

### Detection of urea production

Urea levels in cell culture supernatants were quantified using a microplate-based colorimetric assay. Briefly, samples were incubated with diacetyl monoxime and thiosemicarbazide in acid medium at 37 °C for 40 min. The resulting chromophore was measured at 630 nm, with urea standards (0–50 mg/dL) used to generate a calibration curve. The assay demonstrated a sensitivity of 0.5 mg/dL and linearity across the physiological range (R^2^ > 0.99). Values were normalized to cell number when appropriate. More details about reagents could be found in Table S2.

### Statistical analysis

Intergroup comparisons were performed using Student’s t-test for two-group analyses, while one-way analysis of variance (ANOVA) was applied for multiple group comparisons. All statistical analyses were performed using GraphPad Prism 9. The data shown are representative of at least three independent experiments. Data are presented as the mean ± SD. ns, not significant. *p < 0.05; **p < 0.01; ***p < 0.001; ****p < 0.0001.

## Supplementary Information

Below is the link to the electronic supplementary material.

Supplementary Material 1

## Data Availability

The data underlying this article are available in GEO (Gene Expression Omnibus) at (https://www.ncbi.nlm.nih.gov/geo) and the accession number is GSE308085.
