# Supplementary material for: A robust mouse liver organoid platform enables sustained multicellular maturation and fibrosis modeling from a single tissue sample
Source: Sci Rep. 2026 Mar 19;16:14137. doi: 10.1038/s41598-026-42990-2 (PMC13136479; doi:10.1038/s41598-026-42990-2)
Supplement: Supplementary file 1 — Supplementary Material 1 [file 41598_2026_42990_MOESM1_ESM.pdf]

Table S1. Components of organoids medium

Components	Hepatocyte organoids medium	Cholangiocyte organoids medium
Advanced DMEM/F-12	+	+
1% penicillin/streptomycin	+	+
1% GlutaMAX	+	+
HEPES	10 mM	25 mM
B27 supplement (1:50)	+	+
N2 supplement (1:100)	-	+
R-spondin1	1.5μg/mL	1μg/mL
Noggin	-	100 ng/ml
Wnt3a	-	100 ng/ml
ChIR99021	3μM	-
N-acetylcysteine	1.25mM	1.25mM
nicotinamide	10 mM	10 mM
recombinant gastrin	10 nM	10 nM
recombinant EGF	50 ng/mL	50 ng/mL
recombinant human FGF7	50 ng/ml	-
recombinant human FGF10	50 ng/ml	100 ng/ml
recombinant human HGF	25 ng/ml	50 ng/ml
Forskolin	-	10 μM
A83-01	1mM	5μM
DAPT	10 μM	-
dexamethasone	3 μM	-
ROCK inhibitor (Y-27632)	10 μM	10 μM

Table S2. Resources Table

REAGENT or RESOURCE	SOURCE	IDENTIFIER
Antibodies		
Goat anti-Mouse IgG (H+L) Cross-Adsorbed Secondary Antibody, Alexa Fluor 594	Invitrogen	Cat# A11005; RRID:AB_2534073
Goat anti-Rabbit IgG (H+L) Cross-Adsorbed Secondary Antibody, Alexa Fluor 488	Invitrogen	Cat# A11008; RRID:AB_143165
Anti-Albumin antibody	Abcam	Cat# ab207327; RRID:AB_2755031
Albumin Monoclonal antibody	Proteintech	Cat# 66051-1-Ig; RRID:AB_11042320
Anti-E Cadherin antibody	Abcam	Cat# ab231303; RRID:AB_2923285
ZO-1 Monoclonal antibody	Invitrogen	Cat# 33-9100; RRID:AB_87181
Cytokeratin 19 Polyclonal antibody	Proteintech	Cat# 10712-1-AP; RRID:AB_2133325
AFP Polyclonal antibody	Proteintech	Cat# 14550-1-AP; RRID:AB_2223933
Anti-Cytokeratin 7 antibody	Abcam	Cat# ab181598; RRID:AB_2783822
RBP1 Polyclonal antibody	Proteintech	Cat# 22683-1-AP; RRID:AB_11182381
Desmin Monoclonal antibody	Proteintech	Cat# 67793-1-Ig; RRID:AB_2918557
Bacterial and virus strains		
None		

Biological samples		
None		
Chemicals, peptides, and recombinant proteins		
Advanced DMEM/F-12	Gibco	Cat# 12634028
DMEM, high glucose, HEPES, no phenol red	Gibco	Cat# 21063029
Fetal Bovine Serum	Gibco	Cat# 10500064
HEPES(1M)	Gibco	Cat# 15630-080
GlutaMAX™_(100X)	Gibco	Cat# 35050-061
B27 Supplement 50X	Gibco	Cat# 17504001
N2 Supplement 100X	Gibco	Cat# 17502001
TrypLE Express Enzyme (1X), no phenol red	Gibco	Cat# 12604013
Cellsaving	NCM Biotech	Cat# C40100
specific hepatic stellate cell medium	Procell	Cat# CM-M041
Nicotinamide	Sigma	Cat# N0636
N-Acetylcysteine	Sigma	Cat# A9165
30% Bovine Serum Albumin solution	Sigma	Cat# 9048-46-8
R-spondin1	R&D	Cat# 7150-RS
mEGF	Gibco	Cat# PMG8043
A83-01	stemcell	Cat# 72022
mNoggin	peprotech	Cat# 250-38
Gastrin	Sigma	Cat# G9145

Wnt3a	peprotech	Cat# 31520
FGF10	peprotech	Cat# 100-26
HGF	peprotech	Cat# 100-39
Y27632	Selleckchem	Cat# S1049
FGF7	peprotech	Cat# 100-19
CHIR99021	Tocris	Cat# 4423/10
Dexamethsone	Sigma	Cat# D4902
DAPT	Sigma	Cat# D5942
Rhodamine 123	Sigma	Cat# 83702
+/-Verapamil hydrochloride	Sigma	Cat# V4629
1X RBC Lysis Buffer	Invitrogen	Cat# 00-4333-57
DAPI Solution	Thermo	Cat# 62248
DPBS. no calcium.no magnesium	Gibco	Cat# 14190250
Percoll	Biosharp	Cat# BS909-100ml
CD326 (EpCAM) MicroBeads	Miltenyi	Cat# 130-105-958
Rinsing Solution	Miltenyi	Cat# 130-091-222
BSA Stock Solution	Miltenyi	Cat# 130-091-376
Penicillin/Streptomycin 100X	Gibco	Cat# 15140-122
NaCl	Sangon	Cat# A501218
KCl	Sangon	Cat# A610440-0500
$MgCl_2$. $6H_2O$	Sangon	Cat# A601336-0500
$MgSO_4$. $7H_2O$	Sangon	Cat# A610329-0500
Na_2HPO_4	Sangon	Cat# A501727-0500
KH_2PO_4	Sangon	Cat# A501211-0500
D-(+)-Glucose	Sangon	Cat# A100188-0500
$NaHCO_3$	Sangon	Cat# A100865-0500
$CaCl_2$. $2H_2O$	Sangon	Cat# A610050-0500
DNase I	Stemcell	Cat# 07470-100mg
HBSS	Procell	Cat# PB180323

Nycodenz	Accurate Chemical	Cat# AN1002424
VeZol	Vazyme	Cat# R411-02
iTaq™ Universal SYBR®	Bio-rad	Cat# RR047A
4% PFA	Biosharp	Cat# BL539A
Growth Factor Reduced (GFR) Basement Membrane Matrix, Phenol Red-free, LDEV-free	Corning	Cat# 356231
Human DiO-Low Density Lipoprotein(Human DiO-LDL)	MKBio	Cat# MP6008
Dimethyl sulfoxide	Sigma	Cat# D5879
Fluoromount-G	SouthernBiotech	Cat# 0100-01
HistoGel™ Specimen Processing Gel	Epredia	Cat# HG4000012
PBS (10X)	Gibco	Cat# 70011044
Critical commercial assays		
High Activity General Tissue Enzymatic Digestion Kit	RWD	Cat# DHGT-5004
Mouse Albumin ELISA Kit	Bethyl	Cat# E99-134
PrimeScript™ RT reagent Kit with gDNA Eraser (Perfect Real Time)	Takara Bio	Cat# 1725122
Urea Detection Kit	Solarbio	BC1535
Mouse Cytochrome P450 (CYP450) ELISA Research Kit	Macklin	P770740
Deposited data		
Bulk RNA-seq	This paper	To be uploaded
Smart-seq	This paper	To be uploaded
Experimental models: Cell lines		
None		

Experimental models: Organisms/strains		
BALB/c mice, male	Cyagen BioTech	N/A
Oligonucleotides		
Primer: *Actb* Forward for total mouse cDNA: GGCTGTATTCCCCTCCATCG	This paper	N/A
Primer: *Actb* Reverse for total mouse cDNA: CCAGTTGGTAACAATGCCATG	This paper	N/A
Primer: *Alb* Forward for total mouse cDNA: TCCAAACCTCCGTGAAAACTAT	This paper	N/A
Primer: *Alb* Reverse for total mouse cDNA: TGTGTTGCAGGAAACATTCGT	This paper	N/A
Primer: *Hnf4α* Forward for total mouse cDNA: CACGCGGAGGTCAAGCTAC	This paper	N/A
Primer: *Hnf4α* Reverse for total mouse cDNA: CCCAGAGATGGGAGAGGTGA	This paper	N/A
Primer: *Krt7* Forward for total mouse cDNA: AGGAGATCAACCGACGCAC	This paper	N/A
Primer: *Krt7* Reverse for total mouse cDNA: CACCTTGTTCGTGTAGGCG	This paper	N/A
Recombinant DNA		

None		
Software and algorithms		
GraphPad Prism 9	GraphPad Software Inc	RRID: SCR_002798
Other		
Single Cell Tube	RWD	Cat# SCT-25
LS Separation columns	XinBio	Cat# 92-01-0011
Direct-zol RNA MiniPrep	Zymo Research	Cat# R2052
